# Assessment of the Mutation Profile of Tonsillar Squamous Cell Carcinomas Using Targeted Next-Generation Sequencing

**DOI:** 10.3390/biomedicines11030851

**Published:** 2023-03-10

**Authors:** Ha Young Park, Joong Seob Lee, Jee Hye Wee, Jeong Wook Kang, Eun Soo Kim, Taeryool Koo, Hee Sung Hwang, Hyo Jung Kim, Ho Suk Kang, Hyun Lim, Nan Young Kim, Eun Sook Nam, Seong Jin Cho, Mi Jung Kwon

**Affiliations:** 1 Department of Pathology, Busan Paik Hospital, Inje University College of Medicine, Busan 47392, Republic of Korea; 2 Department of Otorhinolaryngology-Head & Neck Surgery, Hallym University Sacred Heart Hospital, Hallym University College of Medicine, Anyang 14068, Republic of Korea; 3 Department of Radiology, Hallym University Sacred Heart Hospital, Hallym University College of Medicine, Anyang 14068, Republic of Korea; 4 Department of Radiation Oncology, Hallym University Sacred Heart Hospital, Hallym University College of Medicine, Anyang 14068, Republic of Korea; 5 Department of Nuclear Medicine, Hallym University Sacred Heart Hospital, Hallym University College of Medicine, Anyang 14068, Republic of Korea; 6 Department of Hematological Oncology, Hallym University Sacred Heart Hospital, Hallym University College of Medicine, Anyang 14068, Republic of Korea; 7 Department of Internal Medicine, Hallym University Sacred Heart Hospital, Hallym University College of Medicine, Anyang 14068, Republic of Korea; 8 Hallym Institute of Translational Genomics and Bioinformatics, Hallym University Medical Center, Anyang 14068, Republic of Korea; 9 Department of Pathology, Kangdong Sacred Heart Hospital, Hallym University College of Medicine, Seoul 05355, Republic of Korea; 10 Department of Pathology, Hallym University Sacred Heart Hospital, Hallym University College of Medicine, Anyang 14068, Republic of Korea

**Keywords:** oropharynx, palatine tonsil, squamous cell carcinoma, next-generation sequencing, prognosis, TCGA data analysis

## Abstract

Data regarding driver mutation profiles in tonsillar squamous cell carcinomas (TSCCs) remain scarce, limiting the understanding of its pathogenesis and unexpected behavior in the updated staging system. We investigated the incidence of clinically relevant mutations and their contribution in the prognosis of the condition, and their association with human papillomavirus (HPV) infection and adjuvant therapy. We subjected 43 surgically resected TSCC samples to targeted next-generation sequencing, determined their HPV status using polymerase chain reaction, and performed The Cancer Genomic Atlas and Gene Set Enrichment analyses. Thirty-five TSCC samples (81.4%) showed at least one oncogenic/likely oncogenic mutation among twenty-nine cancer-related genes. The top five mutated genes were *TP53* (46.5%), *PIK3CA* (25.6%), *PTEN* (18.6%), *EGFR* (16.3%), and *SMAD4* (14.0%). The EGFR pathway was the most frequently affected (51.2%), followed by the p53 (48.8%), PI3K (39.5%), and RTK (34.9%) pathways. The gene set enrichment analysis confirmed that the genes involved in signal transduction, such as growth factor receptors and second messengers, EGFR tyrosine kinase inhibitors, and PI3K signaling pathways, were mostly related with TSCCs. *TP53* mutation was an independent prognostic factor predicting worse overall survival in the adjuvant therapy group. *RTK* mutations were related to survival in all patients and in the HPV-positive group, but multivariate analyses showed no significance. In conclusion, oncogenic/likely oncogenic mutations were relatively high in TSCCs, and *TP53* and RTK mutations may be candidate predictors for poor prognosis in the adjuvant therapy and HPV-positive groups, respectively, under the updated staging system.

## 1. Introduction

Squamous cell carcinomas (SCCs) of the tonsillar region (TSCCs) are the most prevailing oropharyngeal cancers, constituting 15–20% of all oropharyngeal malignancies and one of the subsites with the highest human papillomavirus (HPV) positivity rate [1,2]. Oropharyngeal cancers are associated with HPV infection, alcohol consumption, smoking, environmental pollution, and genetic risk factors [3]; however, awareness of HPV as a causative agent for these cancers is low [4], resulting in a public health burden [5]. Although the 5-year survival in low-stage cases is >90%, it decreases to <20% in advanced tonsil cancers [1,2,6]. Early detection of tonsil cancer is challenging [5]; the peculiar tonsillar anatomical features make the tumor proliferating beneath the surface mucosa unrecognizable, as do the several lymphatic vessel plexuses present around the tonsils. Thus, the clinical course of TSCC is more aggressive, with significant risk for higher stage and earlier dissemination at diagnosis, as compared to that for other head and neck cancers [7,8].

Combined surgery and radiotherapy/chemotherapy is a standard practice for local or regionally advanced tonsil cancers [9,10] because TSCC is relatively chemo/radiosensitive [7,8,11] and HPV is involved in a more favorable prognosis and response to radiochemotherapy, as compared to those observed in case of HPV-unrelated oropharyngeal cancers [2,10]. In this context, the eighth edition of the American Joint Committee on Cancer (AJCC) TNM classification in 2017 specified HPV-related oropharyngeal SCCs as a separate entity and downstaged this group as compared to the HPV-negative group [12]. However, treatment failure can develop unexpectedly in a loco-regionally controlled disease as therapy-refractory recurrence [13]; 13–25% of patients with HPV-positive oropharyngeal SCCs, including TSCC, develop local/distant recurrence and have a course of disease similar to those of HPV-negative oropharyngeal cancers [14], which may imply potential genetic involvement in tumor behavior and therapeutic drug response at the molecular level [15]. The molecular profiles of tonsil cancers and their clinical or pathological relation under the new eighth-edition AJCC staging system are poorly understood. Therefore, identifying molecular markers under the updated AJCC staging system that are diagnostic and predictive of clinical outcomes may be clinically significant so as to recommend further therapeutic options for potentially responsive patients, thereby improving patient prognosis.

Currently, molecular profiling of various cancers using next-generation sequencing (NGS) has been increasingly implemented in the oncology field for diagnostic and therapeutic applications. A broad range of somatic genetic alterations have been illustrated in head and neck SCCs (HNSCCs), where cancer-specific mutations, including small deletions or insertions and single-nucleotide polymorphisms, are known to influence driver genes during tumor development and progression [15,16,17]. However, despite the increasing prevalence and substantial public health impact of tonsil cancers [18], molecular data that may illustrate the unexpected behaviors or characteristic histological findings of HPV-positive and -negative TSCCs are limited. Owing to the emergence of personalized medicine in conjunction with molecular profiling, there is now a crucial need for detailed research on TSCCs.

Hence, we carried out this retrospective study to detect the frequency of pathogenic mutations in tonsil cancers, to find potential targets for treatment through an NGS cancer panel, and tested whether somatic mutation status or any cancer pathway are associated with risk factors or survival.

## 2. Materials and Methods

### 2.1. Patients and Tumor Samples

The study was based on formalin-fixed, paraffin-embedded (FFPE) tissues acquired from 43 patients with TSCC who underwent primary surgical resection with no prior treatment between 1997 and 2018, and whose complete medical records were available at the present institution. After curative surgery, adjuvant therapy was carried out. In total, 6 patients underwent post-operative radiotherapy, while 21 patients underwent radiochemotherapy following surgical resection. The remaining 16 patients underwent surgery alone. Radiation doses spanned from 5040 to 7200 cGy/36 fractions over eight weeks.

Hematoxylin-and-eosin-stained slides of all cases were examined, and diagnosis and histological characterization were performed by two expert pathologists (MJK and SJC) according to the 2017 World Health Organization classification of head and neck tumors [6]. Patients were re-staged according to the 8th edition of the AJCC TNM classification [12]. Clinical information was analyzed based on medical records and radiological results. Heavy smoking and heavy alcohol consumption were defined as >20 packs/year and >14 drinks/week, respectively [12].

The study protocol was permitted by the Institutional Review Board (IRB no. 2019-06-012) and conducted following the relevant guidelines and regulations (Declaration of Helsinki).

### 2.2. DNA Extraction

Tumor parts on the two 10 μm sections from every FFPE tissue block, on glass slides, were manually macrodissected from the unstained tissue sections to enrich for a tumor cell population of >50%. DNA was extracted and purified using the Ion AmpliSeq™ Direct FFPE DNA Kit (Thermo Fisher Scientific, Waltham, MA, USA) and QIAamp DSP DNA FFPE Tissue Kit (QIAGEN, Hilden, Germany), respectively, as previously described [19]. The yield of purified genomic DNA was measured utilizing a Qubit^®^ 2.0 Fluorometer and Qubit^®^ dsDNA HS Assay Kit (Thermo Fisher Scientific) prior to library preparation for sequencing.

### 2.3. Detection of HPV

HPV status was evaluated using the PANA RealTyper™ HPV Genotyping Kit (PANAGENE, Daejeon, Republic of Korea) approved for clinical use in Korea, identifying a total of 40 HPV genotypes, including 20 high-risk genotypes (16, 18, 26, 31, 33, 35, 39, 45, 51, 52, 53, 56, 58, 59, 66, 68, 69, 70, 73, and 82), two low-risk genotypes (6 and 11), and 18 other genotypes. Real-time polymerase chain reaction (PCR) assays were conducted in a 25 µL reaction mixture consisting of 19 µL of HPV mix, 1 µL of Taq DNA polymerase, and 5 µL of extracted DNA, with positive and negative control, as previously described [20].

### 2.4. Immunohistochemistry

Programmed death-ligand 1 (PD-L1) staining of 4 μm thick tissue sections was performed using the US Food and Drug Administration-approved PD-L1 22C3 pharmDx Kit (Dako North America Inc., Carpinteria, CA, USA) on the Dako Autostainer Link 48, as per the manufacturer’s instructions. The slides were assigned depending on the percentage of positive cells divided by the number of fields, to calculate the average value for each individual case, defined at 200× magnification. The PD-L1 combined positive score was calculated as the ratio of PD-L1-positive cells (tumor or immune cells) to the total number of tumor cells × 100 and classified as positive (≥1) or negative (<1).

### 2.5. Library Preparation and Sequencing

In this study, an Ion Personal Genome Machine (PGM) Sequencer (Thermo Fisher Scientific) was used as the NGS platform. Library preparation for individual samples was accomplished using the Ion AmpliSeq™ Library Kit 2.0 (Thermo Fisher Scientific) and Ion AmpliSeq™ Cancer HotSpot Panel v2 (Thermo Fisher Scientific), as per the manufacturer’s directions [19]. The targeted cancer panel sequences 2790 mutations in 50 oncogenes and tumor suppressor genes with known cancer correlations. Generally, 10 ng of genomic DNA from each sample was applied to prepare barcoded libraries, using Ion Xpress™ Barcode Adapters (Thermo Fisher Scientific). Libraries were pooled to a final concentration of 100 pmol/L using the Ion Library Universal Quantification Kit (Thermo Fisher Scientific), and emulsion polymerase chain reaction was performed utilizing the Ion Torrent™ OneTouch™ 2 System. Every pool was loaded onto an Ion 318v2 Chip (Thermo Fisher Scientific) for single-end sequence analysis with an Ion PGM Sequencer, while applying 500 flows (125 cycles) for 200-base-read sequencing.

### 2.6. Data Analyses, TCGA Data, and Gene Set Enrichment Analysis (GSEA)

Raw sequence reads were mapped against the human reference genome hg19 and cleaned prior to variant calling. Ion Torrent platform-specific pipeline software (Torrent Suite version 5.0.5) was operated throughout the variant calling steps. Annotation was carried out using the VEP and OncoKB™ (an expert-guided precision oncology knowledge base) annotator [21]. To detect confident putative somatic variants, annotated raw variants were filtered using the following criteria: (1) non-synonymous single-nucleotide variant or short insertion or deletion in coding regions; (2) coverage ≥50× and variant allele frequency ≥5%; (3) minor allele frequency <0.1% in gnomAD and 1000 Genomes Project; (4) detected as ‘Oncogenic’ or ‘Likely Oncogenic’ by the OncoKB™ annotator. The resulting list of variants was manually reviewed and visually confirmed using the Integrated Genomics Viewer (http://www.broadinstitute.org/igv/; accessed on 15 June 2020). R and MutationMapper (https://www.cbioportal.org/mutation_mapper, accessed on 10 January 2023) were used for visualization.

To compare the data with those of previous cancer genome studies on tonsil cancer and verify hotspot mutations, we downloaded the ‘Head and Neck Squamous Cell Carcinoma from the Cancer Genome Atlas (TCGA, PanCancer Atlas)’ (TCGA HNSC) (http://cancergenome.nih.gov/abouttcga (accessed on 5 September 2022)) sequence variant dataset in mutation annotation format (MAF) from cBioPortal (https://www.cbioportal.org/) (accessed on 5 September 2022). We performed OncoKB™ annotation on the TCGA HNSC MAF file and filtering using the same criteria as described above for comparison under the same conditions as our data.

The 29 altered genes identified in this study were subjected to *GSEA* [22] using the canonical pathways of the Molecular Signatures Database (http://www.gsea-msigdb.org/gsea/msigdb/human/annotate.jsp; accessed on 10 January 2023), including BIOCARTA, KEGG, PID, REACTOME, and WIKIPATHWAYS, which determine pathway correlations with major biomolecular and cellular procedures [23]. For genes that could be assigned to multiple pathways, we chose the signaling with the highest fraction of mapped genes. Additionally, knowledge of the functional classification of the major signaling molecules in the chosen pathway was used. A result was considered significant if the cut-off of the false discovery rate was ≤ 0.01 and *p*-value was < 0.05.

### 2.7. Statistical Analyses

Statistical analyses of the data for pairs of groups were conducted using the Fisher’s exact test for categorical variables and Wilcoxon rank-sum test for continuous variables. Survival curves were produced using the Kaplan–Meier method, and a log-rank test was performed to calculate the statistical significance between groups. Overall survival (OS) was clarified as the interval from the first day of surgery until death. Disease-free survival (DFS) was described as the interval from the first day of surgery until tumor recurrence. The OS and DFS were analyzed until February 2019. Univariate and multivariate analyses applying the Cox proportional hazard regression model were used to measure the hazard ratios (HRs) and 95% confidence intervals (CIs) for specific variables involved in OS and DFS. The prognostic variables (*p* < 0.1) observed using univariate analysis by means of the log-rank test were additionally analyzed using multivariate analysis. A two-sided *p* < 0.05 was considered to indicate a statistically significant difference. Statistical analyses were carried out using Rex software (version 3.3.1, RexSoft Inc., Seoul, Republic of Korea).

## 3. Results

### 3.1. Patient Characteristics

The clinical and pathological features of the patients with TSCC are outlined in Table 1. Of the 43 patients, 38 (88.4%) were men and 5 (11.6%) were women, with an average age at diagnosis of 54 years (range, 36–80 years). Twenty-one (50.0%) patients were heavy smokers, while fifteen (35.7%) were heavy alcohol drinkers. Only high-risk HPV genotypes were identified in 35 (81.4%) of the 43 patients—HPV 16 was detected in 28 (80.0%), HPV 18 in 4 (11.4%), and HPV 16 and 18 occurred concurrently in 3 (8.6%) patients.

As per the 8th AJCC staging system, 9 (20.9%) tumors were categorized as T1, 13 (30.25%) as T2, 13 (30.25%) as T3, and 8 (18.6%) as T4; 8 (18.6%) were categorized as N0, 18 (41.9%) as N1, 8 (18.6%) as N2, and 9 (20.9%) as N3. Upon merging the T and N categories, the overall stage of 15 patients was diagnosed as stage I (34.9%), 8 (18.6%) as stage II, 7 (16.3%) as stage III, and 13 (30.2%) as stage IV. All 43 patients with tonsil cancers were treated with surgical resection, of which 28 (65.1%) additionally received post-operative chemotherapy and/or radiotherapy. The median follow-up period was 56 months (range, 16–121 months), with 5-year OS and DFS rates of 55.8% and 48.8%, respectively.

### 3.2. Molecular Profiling in Overall TSCCs

**Figure 1:**
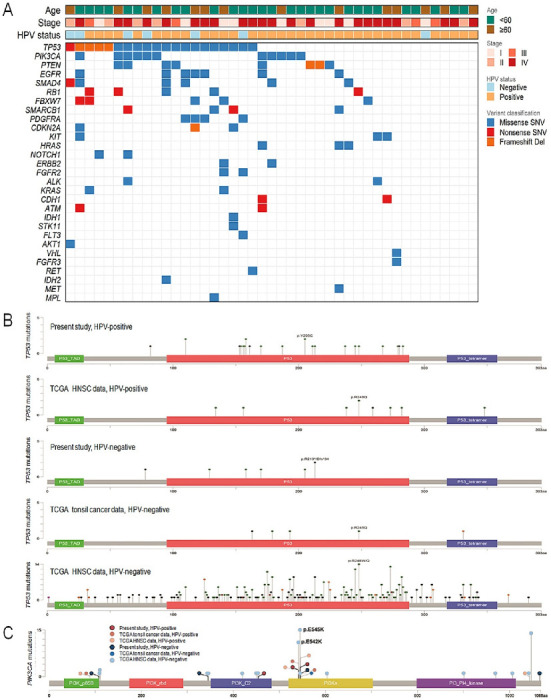
(**A**) Schematic overview of the overall mutation profile of 43 tonsil cancers, followed by targeted next-generation sequencing and analysis. Each column represents a case. The top three panels show the age, stage, and HPV status. The bottom panel shows the distribution of mutations. The four mutation types have been distinguished using different colors. The right panel represents the comparison of mutation frequencies among our cases, overall head and neck cancers, and tonsil cancers retrieved from the TCGA data. Locations of *TP53* (**B**) and *PIK3CA* (**C**) mutations found in tonsillar squamous cell carcinomas. A circle represents the presence of a single mutation case. Deep-red- and dark-blue/black-lined circles indicate cases in this study with and without HPV infection, respectively; others have been retrieved from the TCGA tonsil cancer and TCGA HNSC data. The line length depends on the number of mutations detected in that codon. The colored boxes are specific functional domains. Above the lollipops, the frequent variants have been annotated as the amino acid change at that particular site. HPV, human papillomavirus; TCGA, The Cancer Genomic Atlas.

The genetic landscape of all the cases is summarized in Figure 1A. On average, there were 405,114 reads (range 128,509–711,217) per case. An average of 96.4% of the reads (range 81.9–99.6%) per case was mapped to the intended targeted areas of the human genome. All regions had an average coverage of 1531× (range 163×–3417×). After the multiple filtering process described in the Materials and Methods section, we identified 120 variant calls of validated mutations for 29 genes in 35 cases (81.4%) as oncogenic/likely oncogenic mutations in the OncoKB™ database, including 98 (81.7%) missense point mutations (single-nucleotide variants) in 32 cases, 13 (10.8%) nonsense mutations in 9 cases, and 9 (7.5%) frameshift deletion mutations in 8 cases (Table S1). A total of 26 of the 43 cases (60.5%) harbored concurrent mutations in two or more genes.

**Figure 2:**
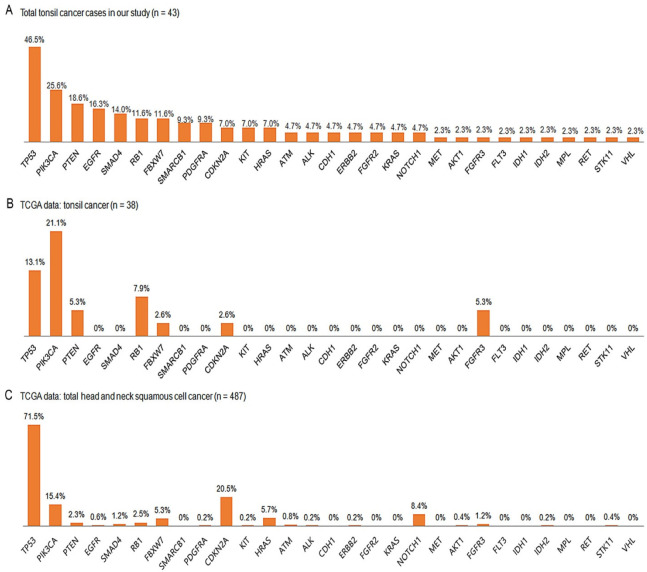
Schematic comparison of mutation frequencies between the cases in our cohort (**A**) and TCGA data (tonsil cancer (**B**) and total head and neck squamous cell cancer (**C**)). TCGA, The Cancer Genomic Atlas.

Among the mutated genes, *TP53* mutations were most recurrent, in 20 of the 43 (46.5%) patients, followed by *PIK3CA* in 11 (25.6%), *PTEN* in 8 (18.6%), *EGFR* in 7 (16.3%), and *SMAD4* in 6 (14.0%) patients (Figure 2A). *RB1* and *FBXW7* variants were observed in 5 of 43 (11.6%) patients each, followed by *SMARCB1* and *PDGFRA* variants in 4 each (9.3%), and *CDKN2A*, *KIT*, and *HRAS* variants in 3 each (7.0%). *ATM*, *ALK*, *CDH1*, *ERBB2*, *FGFR2*, *KRAS*, and *NOTCH1* variants were found in 2 of 43 (4.7%) patients each, followed by *MET*, *AKT1*, *FGFR3*, *FLT3*, *IDH1*, *IDH2*, *MPL*, *RET*, *STK11*, and *VHL* variants in a single case each (2.3%).

As per OncoKB™, 14 genes (*ALK*, *ATM*, *ERBB2*, *EGFR*, *FGFR3*, *FLT3*, *IDH1*, *IDH2*, *KIT*, *KRAS*, *MET*, *PDGFRA*, *PIK3CA*, and *SMARCB1*) having level 1 evidence were observed in 25 (58.1%) patients.

The *TP53* mutations (*n* = 30) were present in exons 4–8, including the following (Figure 1B): p.R283H (*n* = 1), p.R280Efs*65 (*n* = 1), p.G279V (*n* = 1), and p.R267Q (*n* = 1) in exon 8; p.R248W (*n* = 1), p.G245D (*n* = 1), and p.M237I (*n* = 1) in exon 7; p.Y205C (*n* = 3), p.R213Dfs*34 (*n* = 1), p.R213*(*n* = 1), p.R213Q (*n* = 1), p.T211I (*n* = 1), and p.G187V (*n* = 1) in exon 6; p.T170A (*n* = 1), p.T170M (*n* = 1), p.A161Pfs*9 (*n* = 1), p.R158H (*n* = 1), p.R158C (*n* = 2), p.V157I (*n* = 1), p.R156C (*n* = 1), p.G154V *(n* = 1), p.P153Afs*16 (*n* = 1), and p.A129V (*n* = 1) in exon 5; and p.R110C (*n* = 2), p.P82Rfs*41 (*n* = 1), and p.A78Qfs*45 (*n* = 1) in exon 4. Only 6.7% of *TP53* missense mutations were observed in codons R248, R273, G245, R175, R282, and H179, which are the most recurring hotspot variants in HNSCCs [24].

The p.R158 (*n* = 3), p.R213 (*n* = 3), or p.Y205 (*n* = 3) variants of *TP53* mutations appeared in more than two cases, while the c.614A > G (p.Y205C) transversion variant occurred in three patients. In contrast, *PIK3CA* hotspot variants (E542, E545) were identified in 20.9% (9/43) of TSCC patients, with four samples (9.3%) bearing non-hotspot mutations (p.R108H, p.V344M, p.E453K, and p.A1066T). Other *PIK3CA* hotspot mutations such as p.H1047 were not identified. The most frequent alterations in *PIK3CA* were E545K and p.E542K mutations (69.2%) (Figure 1C).

Genetic alterations in coding sequences, including missense, frameshift, and nonsense mutations, were categorized into several gene pathways and groups on the basis of the known function of the genes (Table 2). In TSCCs, the epidermal growth factor receptor (EGFR) pathway, including alterations in *EGFR* and downstream genes (*ERBB2*, *KRAS*, *HRAS*, *PIK3CA*, *PTEN*, and *AKT1*), was the most frequently affected, in 22 (51.2%) cases, followed by the p53 pathway, including alterations in *TP53* and *ATM*, in 21 (48.8%), phosphatidylinositol 3-kinase (PI3K) pathway in 17 (39.5%), receptor tyrosine kinase (RTK) pathway in 15 (34.9%), retinoblastoma tumor suppressor (RB) pathway in 8 (18.6%), transforming growth factor-beta (TGF-β) pathway in 6 (14.0%), mitogen-activated protein kinase (MAPK) pathway in 5 (11.6%), proteolysis pathway in 5 (11.6%), and SWItch/Sucrose Non-Fermentable complex pathway in 4 (9.3%) cases. Other signaling pathways, including Notch, Hippo, mammalian target of rapamycin, metabolism, and transcription factors/regulators, had mutations with minor frequencies.

### 3.3. Comparisons between HPV-Positive and -Negative TSCCs

We next assessed the differential genetic alterations in HPV-positive and -negative tonsil cancers. Among the 120 variant calls of the 50 evaluated genes, HPV-positive tumors carried a meaningfully higher frequency of mutations than HPV-negative tumors (73.3% (88/120) vs. 26.7% (32/120), respectively).

**Figure 3:**
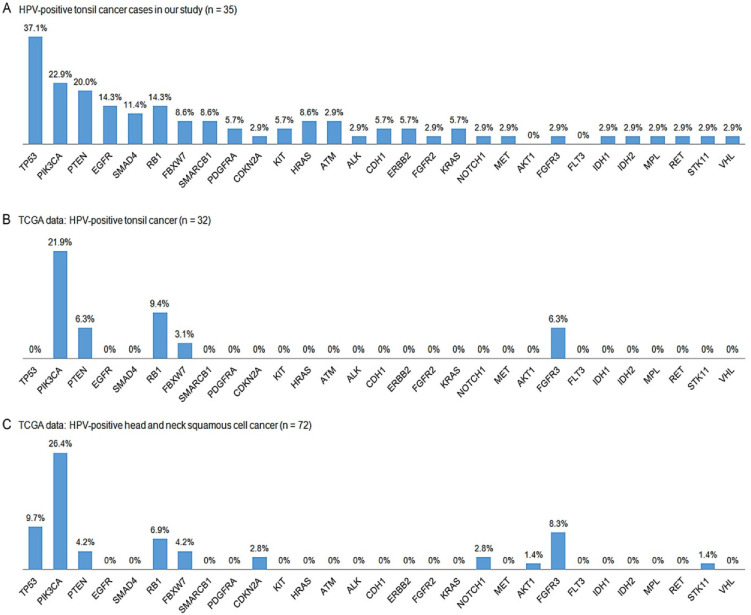
Schematic comparison of each mutation frequency between HPV-positive tonsil cancers among the cases in our cohort (**A**) and TCGA data (tonsil cancers (**B**) and total head and neck cancers(**C**)). TCGA, The Cancer Genomic Atlas; HPV, human papillomavirus.

The most repeated mutation in the HPV-positive tumors was in *TP53* (37.1%, 13/35), followed by those in *PIK3CA* (22.9%, 8/35), *PTEN* (20.0%, 7/35), *EGFR* and *RB1* (14.3%, 5/35 each), and *SMAD4* (11.4%, 4/35) (Figure 3A). The EGFR pathway had the most frequently altered signaling in HPV-positive tumors (48.6%, 17/35), followed by the p53 (40.0%, 14/35), PI3K (37.1%, 13/35), RTK (31.4%, 11/35), RB and TGF-β (17.1%, 6/35 each), and Notch (8.6%, 3/35) pathways.

**Figure 4:**
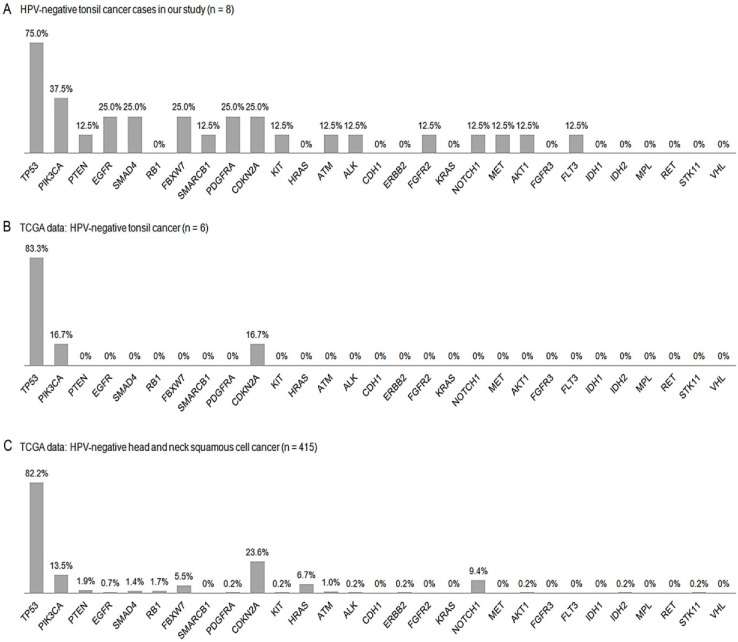
Schematic comparison of each mutation frequency between HPV-negative tonsil cancers among the cases in our cohort (**A**) and TCGA data (tonsil cancers (**B**) and total head and neck cancers(**C**)). TCGA, The Cancer Genomic Atlas; HPV, human papillomavirus.

The most recurrent mutation in HPV-negative tumors was in *TP53* (87.5%), followed by those in *PIK3CA* (37.5%) and *EGFR*, *FBXW7*, *PDGFRA*, and *CDKN2A* (25.0% each) (Figure 4A). The most frequently altered pathway was the p53 pathway (87.5%) in HPV-negative tumors, followed by the EGFR (62.5%, 5/8) and PI3K and RTK pathways (50.0%, 4/8 each).

We further examined whether specific somatic mutations might be related to the discordant cases between the p16 immunohistochemistry and HPV PCR results. We found 10 discordant cases, consisting of HPV-DNA-detected but p16-negative tumors (*n* = 8) and HPV-DNA-negative but p16-positive tumors (*n* = 2). Among the somatic mutations in the 29 genes detected in this study, the *SMARCB1* mutation tended to be more frequently detected in the discordant cases than in the concordant ones (*p* = 0.020).

### 3.4. Impact of Gene Mutations and Pathway Mutations on Clinicopathological Features

Next, we assessed the influence of important gene mutations and the primarily affected gene pathways in tonsil cancers on clinicopathological features (Table 3). Significantly more patients with *TP53* mutations were heavy smokers (*p* = 0.033), HPV-negative (*p* = 0.017), node-negative (pN0; *p* = 0.038), and PD-L1-negative (*p* = 0.011), as compared to those without. *CDKN2A* mutations were considerably correlated with a history of heavy alcohol consumption (*p* = 0.045) and node-negative tumors (*p* = 0.014) (data not shown). *FBXW7* mutations were correlated with a history of heavy smoking (*p* = 0.048) (data not shown). With the exceptions of the *TP53*, *CDKN2A*, and *FBXW7* mutations, no other associations were detected between a single somatic mutation and the evaluated clinicopathological variables. 

### 3.5. Prognostic Genetic Factors in Overall Tonsil Cancers

**Figure 5:**
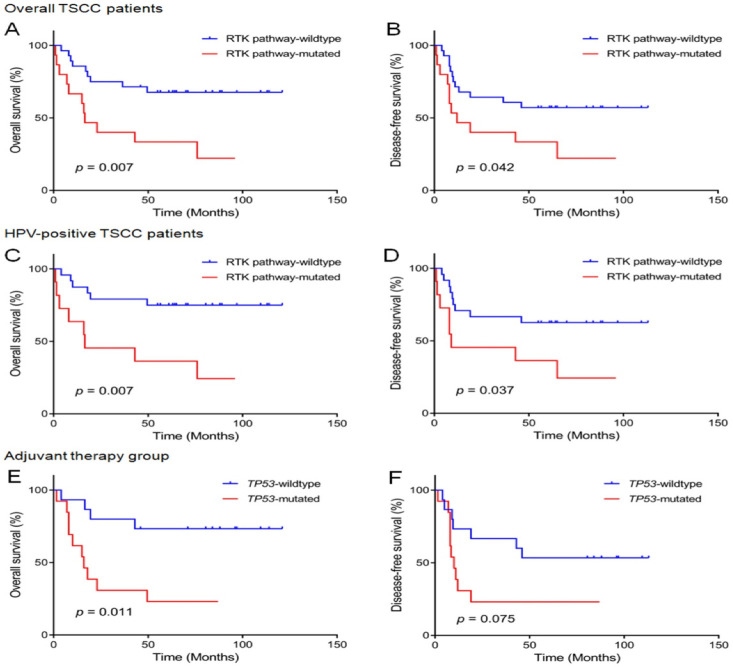
Overall (**A**) and disease-free (**B**) survival by RTK pathway gene mutation status in patients with tonsil cancer who underwent curative surgery. The presence of RTK pathway gene mutations is strongly associated with shorter overall and disease-free survival. In HPV-positive tonsil cancers, RTK pathway gene mutations are associated with worse overall (**C**) and disease-free (**D**) survival. Overall (**E**) and disease-free (**F**) survival by *TP53* mutation status in patients who underwent curative surgery and adjuvant therapy. The *TP53* mutation strongly correlates with decreased overall survival in patients following resection and adjuvant therapy. HPV, human papillomavirus; RTK, receptor tyrosine kinase.

We analyzed the prognostic relationships of genetic alterations and clinicopathological features with OS and DFS in patients with TSCC (Table 4). With respect to OS (Figure 5A), RTK pathway mutations (*p* = 0.007), older age (>60 years; *p* = 0.011), and advanced disease stage (*p* = 0.003) were associated with shorter patient survival. Factors with *p* < 0.1 in the univariate analysis were incorporated into the subsequent multivariate analysis. Older age (*p* = 0.020, HR = 3.565, 95% CI = 1.221–10.412) and advanced stage (*p* = 0.006, HR = 5.246, 95% CI = 1.596–17.235) were independent predictors of shorter OS in patients with TSCC; however, RTK pathway mutations did not reach statistical significance in the multivariate analysis. Kaplan–Meier survival analyses showed that patients with HPV-positive TSCCs had significantly better OS rates than those with HPV-negative TSCCs (mean 80 months vs. 25 months; *p* = 0.049); however, the difference was not statistically significant and showed only borderline significance in the univariate analysis carried out using the Cox proportional hazard regression model (*p* = 0.058).

In contrast, patients with either RTK pathway mutations (*p* = 0.042) or advanced stage disease (*p* = 0.008) exhibited worse prognostic outcomes in terms of DFS (Figure 5B). RTK pathway mutations failed to reach statistical significance in the multivariate analysis. Only advanced stage was an independent negative prognostic factor influencing DFS (*p* = 0.029, HR = 2.856, 95% CI = 1.110–7.345) in the multivariate analyses.

### 3.6. Prognostic Genetic Factors in HPV-Positive TSCCs

We further surveyed prognostic factors related to OS and DFS in HPV-positive tonsil cancers (Table 5). In the univariate analyses, RTK pathway mutations (Figure 5C,D), older age (>60 years), and advanced stage were related with shorter OS and DFS rates (*p* = 0.007, 0.003, and 0.006, respectively, for OS; *p* = 0.037, 0.021, and 0.009, respectively, for DFS). Multivariate analysis showed that older age (>60 years) and advanced stage were independent prognostic factors for worse OS and DFS (*p* = 0.002, HR = 10.521, 95% CI = 2.426–45.616; *p* = 0.003, HR = 9.576, 95% CI = 2.141–42.823, respectively, for OS; *p* = 0.010, HR = 4.378, 95% CI = 1.429–13.408; *p* = 0.007, HR = 4.700, 95% CI = 1.520–14.530, respectively, for DFS); however, RTK pathway mutations did not reach statistical significance in the multivariate analysis for HPV-positive tonsil cancers.

### 3.7. Prognostic Genetic Factors in Patients Who Received Adjuvant Therapy following Surgery

We next investigated the prognostic potential of genetic alterations in patients with TSCC who underwent adjuvant therapy following surgical resection (Table 6). *TP53* mutations (*p* = 0.011), p53 pathway mutations (*p* = 0.029), and advanced stage (*p* = 0.017) were highly correlated with decreased OS in these patients (Figure 5E). Multivariate analyses revealed that *TP53* mutation (*p* = 0.022, HR = 4.348, 95% CI = 1.242–15.223) and higher stage (*p* = 0.024, HR = 4.856, 95% CI = 1.228–19.201) were independent prognostic factors predicting a decrease in OS in those who received adjuvant therapy.

With respect to DFS, *TP53* mutations showed borderline statistical significance in affecting DFS in both the univariate (*p* = 0.075) and multivariate (*p* = 0.059) analyses (Figure 5F). Advanced stage was the only independent poor prognostic factor for DFS in patients who underwent resection with adjuvant therapy (*p* = 0.039, HR = 3.075, 95% CI = 1.057–8.944).

### 3.8. Comparisons with TCGA Data

We compared our results with TCGA data for validation purposes. When we exclusively sorted tonsil cancer data from the TCGA database, we found only 38 cases of tonsil cancer, which were fewer than the number of cases in our study. Owing to this small number, we unavoidably had to retrieve 487 cases of overall HNSC from the TCGA database.

In the TCGA tonsil cancer data (*n* = 38), only seven gene mutations (in *TP53*, *PIK3CA*, *PTEN*, *RB1*, *FBXW7*, *CDKN2A*, and *FGFR3*) have been reported (Figure 2B). *TP53* and *PIK3CA* mutations were the two most common variants, which is consistent with the results of our study; the frequency of *TP53* mutations (13.1%) was lower than that found in our results (46.5%). In the TCGA HNSC data (*n* = 487), *TP53* mutations were the most common (71.5%), followed by *CDKN2A* (20.5%), *PIK3CA* (15.4%), *NOTCH1* (8.4%), *HRAS* (5.7%), *FBXW7* (5.3%), *RB1* (5.3%), and *PTEN* (2.3%) variants (Figure 2C), which were identified in our study as well.

The HPV positivity (32/38, 84.2%) noted in the TCGA tonsil cancer data (Figure 3B) was highly comparable to the 81.4% HPV positivity observed in our cohort (35/43). Upon comparing the HPV positivity of all TCGA HNSC dataset samples (72/487, 14.8%) (Figure 3C), the present study found that the tonsil cancers showed much higher HPV positivity. The HPV negativity (6/38, 15.8%) observed in the TCGA tonsil cancer data (Figure 4B) was also similar to the 18.6% HPV negativity in our cohort (8/43). In the TCGA tonsil cancer and TCGA HNSC data, *TP53* mutations were the most common (83.3% and 82.2%, respectively), followed by *PIK3CA* (16.7% and 13.5%, respectively) and *CDKN2A* (16.7% and 23.6%, respectively) (Figure 4C) mutations, comparable to the findings in our cohort (75.0%, 37.5%, and 25.0%, respectively). In our HPV-positive TSCC data, most *PIK3CA* mutations were E542/545K (7/8, 87.5%), whereas this was much lower in the HPV-negative group (2/5, 40%). Similar results were also observed in TCGA HNSCC dataset, 81.8% (18/22) for the HPV-positive and 50.0% (32/64) for the HPV-negative group. TCGA tonsil cancer showed the same mutation rate in the HPV-positive group (7/8, 87.5%) and only one *PIK3CA* mutation (E545K) was noted in the HPV-negative group.

In the present study, there were differences in *RB1* (14.3% vs. 0%), *PDGFRA* (5.7% vs. 25.0%), *CDKN2A* (2.9% vs. 25.0%), *HRAS* (8.6% vs. 0%), *NOTCH1* (2.9% vs. 12.5%), *AKT1* (0% vs. 12.5%), *FGFR3* (2.9% vs. 0%), and *FLT3* (0% vs. 12.5%) expression upon comparing HPV-positive tonsil cancers with HPV-negative tonsil cancers, respectively. HPV-positive tumors tended to have higher rates of *RB1*, *HRAS*, and *FGFR3* expression, whereas HPV-negative tumors displayed a higher tendency for *PDGFRA*, *CDKN2A*, *NOTCH1*, *AKT1*, and *FLT3* expression.

In HPV-associated and -unrelated TCGA tonsil cancer data, the tendency for *CDKN2A* (0% vs. 16.7%, respectively), *FGFR3* (6.3% vs. 0%, respectively), and *RB1* (9.4% vs. 7.9%, respectively) expression was comparable to that observed in our cohort. However, reports regarding *PFGFRA*, *HRAS*, *AKT1*, *FLT3*, and *NOTCH1* are lacking in the TCGA tonsil cancer data. Upon additionally analyzing TCGA HNSC data, *PDGFRA* (0% vs. 0.2%, respectively) and *NOTCH1* (2.8% vs. 8.4%, respectively) showed a trend that was similar to our results, whereas *HRAS* (0% vs. 5.7%, respectively), *AKT1* (1.4% vs. 0.4%, respectively), and *FLT3* (0% vs. 0%, respectively) showed lower expression.

**Figure 6:**
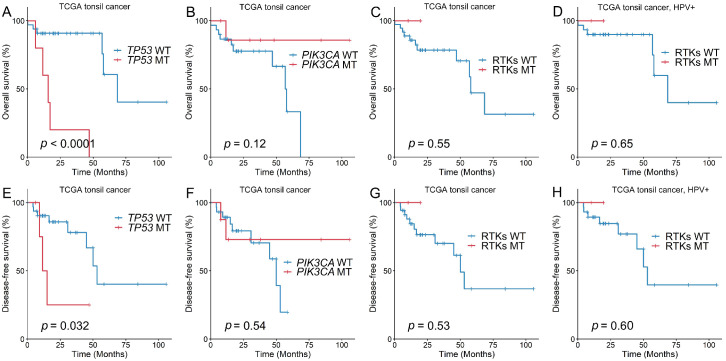
Overall (**A**–**D**) and disease-free (**E**–**H**) survival rates in the TCGA tonsil cancer data, depending on the mutation status of *TP53*, *PIK3CA*, and *RTK*. TCGA, The Cancer Genome Atlas.

Survival analyses of TCGA tonsil cancer data were performed in accordance with the mutation status of *TP53*, *PIK3CA*, and *RTK* pathway genes (Figure 6). *TP53* mutations had worse prognostic consequences for OS (*p* < 0.0001) and DFS (*p* = 0.032) in the TCGA tonsil cancer cohort. *PIK3CA* and RTK pathway gene mutations, on the other hand, were not correlated with OS or DFS in the TCGA tonsil cancer cohort, even in the HPV-positive group.

### 3.9. Identification of Gene Signatures Using GSEA

**Figure 7:**
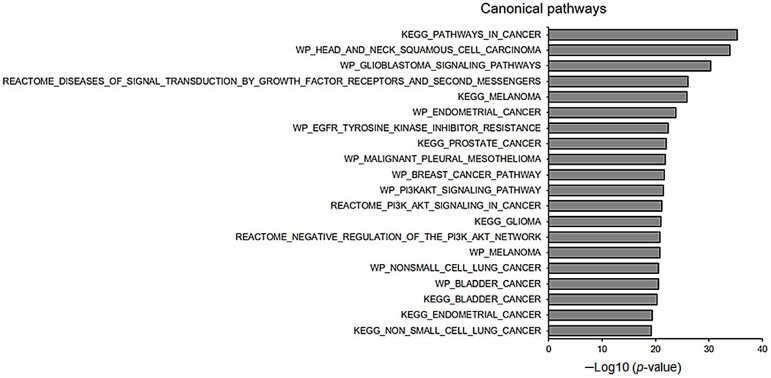
Enriched pathways for the 29 genes with identified mutations upon assessment using gene set enrichment analysis.

We then carried out a GSEA with the identified mutations of the 29 genes. The top 20 enriched gene sets are shown in Figure 7. Several malignancy-related gene sets, including head and neck SCCs, glioblastoma, melanoma, endometrial cancer, prostate cancer, and breast cancer, were strongly enriched. The genes involved in signal transduction, such as growth factor receptors and second messengers, EGFR tyrosine kinase inhibitors, and PI3K signaling pathways, were mostly related to the 29 genes observed in TSCCs.

## 4. Discussion

In our study, somatic mutations were relatively common in tonsil cancers, of which 81.4% had one or more oncogenic/likely oncogenic mutations in twenty-nine cancer-related genes, and 48.6% displayed concurrent mutations in two or more genes. *TP53* (46.5%), *PIK3CA* (25.6%), *PTEN* (18.6%), *EGFR* (16.3%), and *SMAD4* (14.0%) were the top five mutated genes. The fact that these genes are the most frequently altered genes in HNSCC, including TSCCs, represents a critical therapeutic target in patients with TSCCs [17,25,26,27]. In this study, the presence of *TP53* mutations was highly correlated with reduced OS and DFS in patients who underwent adjuvant treatment following surgical resection, and multivariate analysis disclosed it to be an independent factor associated with inferior OS. As the *TP53* gene is widely inactivated for its tumor-suppressive function in a number of cancers, and *TP53* mutation has been reported to confer resistance to cancer therapies, its reactivation or restoration may be a highly attractive therapeutic target for treating the disease, resulting in tumor regression [28]. Unfortunately, this trial is generally regarded as difficult and has only been demonstrated in animal and cell line models [29,30,31]. Synergistic effects have been displayed in the TSCC cell line when complementing single PI3K, PARP, and WEE1 inhibitor medications with 10 Gy or combining these inhibitors [31]. In a current Phase I clinical trial, the WEE1 kinase inhibitor adavosertib (AZD1775) has exhibited beneficials effects on *TP53* mutants in the context of HNSCC [32].

The pathways involving EGFR signaling (51.2%), p53 signaling (48.8%), PI3K (39.5%), and RTKs (34.9%) were found to be frequently involved, suggesting that they may contribute to the tumorigenesis and progression of tonsil cancer [11,27,33,34,35,36,37]. These mutations were also identified in the TCGA HNSC data; however, TCGA tonsil cancer data were available for only a small number (*n* = 38) of patients with tonsil cancer, which is a smaller patient cohort than that in our study, thus reflecting the importance of our research. In our GSEA, the 29 genes detected in TSCCs were confirmed to be linked with the gene signatures in signal transduction by growth factor receptors and second messengers, EGFR tyrosine kinase inhibitors, and PI3K signaling pathways. In the present study, RTK pathway mutations, including *EGFR*, were considerably associated with reduced OS and DFS upon univariate analyses, in the overall TSCCs as well as the HPV-positive subgroup; however, multivariate analyses did not reach statistical significance in this case. Overall, RTK somatic mutations, including *EGFR* and *MET*, mediate therapeutic resistance in HNSCC [33]. However, the prognostic and predictive relevance of individual *RTK* mutations are not clear in HNSCC, including tonsil cancer [33]. As these subsets of patients may potentially benefit from a drug targeting the related pathways, effective screening of molecular-targeted genes may be of great clinical relevance. Targeting *EGFR* utilizing anti-EGFR monoclonal antibodies or kinase domain inhibitors with concomitant radiation is a treatment choice for patients with HNSCC [38]. Several tyrosine kinase inhibitors, such as gefitinib, erlotinib, and afatinib, have also been used to treat HNSCC [39].

*TP53* and *PIK3CA* mutations were the top two common alterations in tonsil cancer, independent of HPV infection status. We found no relationship between clinicopathological variables and *PIK3CA* mutation in tonsil cancer. The lack of specific clinicodemographic features concerned with *PIK3CA* mutation has been suggested in oropharyngeal cancers [27], although some studies have characterized rare patients with decreased exposure to tobacco or HPV-positivity by the clinicopathological features of *PIK3CA* mutation [16,40]. Previous studies have reported that *TP53* mutations are common in HPV-negative tumors, while *PIK3CA* mutations are frequent in HPV-positive cancers [27,40,41]. Recent NGS-based studies identified *TP53* (9.3–25.0% vs. 63.8–85.7%) and *PIK3CA* (20.1–21.4% vs. 6.4–32.1%) in HPV-positive and -negative oropharyngeal SCCs [42], respectively, indicating that although the total number of *TP53* and *PIK3CA* mutations may differ depending on the HPV status, these may still be relatively prevalent [16,42]. Meanwhile, HPV-positive and -negative tonsil cancers may likely have different preferences for cancer-related pathways and detailed somatic mutations: HPV-positive tumors tend to have higher rates of *RB1*, *HRAS*, and *FGFR3* mutations. Of note, *FGFR3* has been identified as a common variant, particularly in HPV-positive tonsil/base of tongue cancers, where *FGFR3* mutations have indicated worse prognosis of patients [27]. Similarly, in our study, RTK mutations, including *FGFR3*, were associated with low OS and DFS in the HPV-positive group upon univariate analysis. In contrast, HPV-negative tumors showed a higher tendency for *TP53*, *FBXW7*, *PDGFRA*, *CDKN2A*, *NOTCH1*, *AKT1*, and *FLT3* mutations in the present study. Reder et al. [42] also demonstrated that HPV-negative oropharyngeal cancers have a significantly higher frequency of mutations in *TP53*, *FAT1, KRAS, NOTCH1*, and *NRAS*, with the reported *FBXW7* (25.0%) mutation frequency being consistent to that observed in our study. *TP53*, *CDKN2A*, and *NOTCH1* have repeatedly been reported to be associated with HPV-negative oral/oropharyngeal SCCs [16,42]. Likewise, the clinicopathological correlation of our study suggested that *TP53*, *CDKN2A*, and *FBXW7* mutations may be closely linked with HPV-negative smoking/alcohol-consumption-related tumors; *TP53* mutation was associated with heavy smoking, HPV-negativity, node-negativity (pN0), and PD-L1-negativity; *CDKN2A* mutation with heavy alcohol drinking history and node-negative tumors; and *FBXW7* mutation with heavy smoking history. Mutations in *CDKN2A* and *TP53* have been described among the earliest genetic events in oral dysplastic lesions [15], which may more likely indicate HPV-negative tumors. Notably, there are no precursor lesions of HPV-associated oropharyngeal SCCs [5,43].

The eighth edition of the AJCC staging system for oropharyngeal cancer has been updated with the incorporation of HPV infection and extranodal extension [12], and now offers better stratification between early or advanced stages and T or N categories for OS than the seventh edition for tonsil cancers [20]. However, the prognostic implication of the various somatic mutations in tonsil cancers has rarely been reported, with little information on staging under the eighth edition of the AJCC guidelines [41,42]. Under this updated staging system, we identified the AJCC stage as the best independent prognostic indicator in tonsil cancers, regardless of HPV status or adjuvant therapy. RTK pathway mutations were likely associated with adverse prognostic impact on OS and DFS in patients with TSCC, although they were not an independent prognostic factor. Nevertheless, RTK pathway gene mutations were more clinically relevant to the advanced stage of tonsil cancers than other mutations in this study. With respect to HPV, HPV infection did not carry an independent prognostic implication for OS and DFS in our study, which may be due to the notable contribution of HPV to the T and N categories of the staging system. The impact of the staging modification might have obscured the effect of HPV because our study analyzed all HPV-positive as well as -negative tumors [20].

*TP53* mutations, despite having the highest frequency (46.5%), occurred at only 6.7% of the hotspot codons in our study, indicating that most *TP53* mutations are widely distributed across exons 4–8 in non-hotspot positions in tonsil cancers. *PIK3CA* variants, the second most common (25.6%) in TSCCs, were mainly located in non-hotspot regions, and their hotspot mutations (E542, E545, and H1047) only accounted for one-fifth of *PIK3CA* variants. This may emphasize the need for caution in the production of sequencing primers and their interpretation when attempting identification using direct sequencing. Earlier studies using direct sequencing and hotspot-targeted primers have reported low rates of *TP53* (21–23%) [34,44] and *PIK3CA* (16–19.5%) mutations in oral/oropharyngeal SCCs [36,40,45]. Instead, a comprehensive NGS panel may be effective in assessing a broad spectrum of genetic alterations in TSCCs. Recent studies on oropharyngeal cancers using NGS platforms have reported high *TP53* (33–55.4%) and *PIK3CA* (26.8%) mutation frequencies, comparable to those observed in our study [41,42,46]. Notably, 58.1% of tonsil cancers harbored candidate target mutations in 14 genes (*ALK*, *ATM*, *EGFR*, *ERBB2*, *FGFR3*, *FLT3*, *IDH1*, *IDH2*, *KIT*, *KRAS*, *MET*, *PDGFRA*, *PIK3CA*, and *SMARCB1*) with level 1 evidence in OncoKB™, which could be implicated in targeted therapies in the future. In addition, we found that *SMARCB1* mutations were more frequent in the 10 discordant cases (23.3%) between p16 immunohistochemistry and HPV PCR results. Although the significance of discordant cancers is controversial, patients with discordant cases demonstrate a borderline significant trend toward survival differences compared to those with concordant cases, which may be a potential therapeutic strategy for discrepant tonsil cancers with *SMARCB1* mutation [47,48,49]. This may also imply that NGS-based gene analyses of tonsil cancer may potentially contribute to providing comprehensive treatment options, including molecularly targeted therapies; however, in the current scenario, NGS testing is not yet routine.

One of the limitations of this study is that we used a limited cancer-related gene panel that lacked information on copy number alterations. Another limitation is the small number of tonsil cancers included in the NGS analyses. Nevertheless, our study improved the understanding of mutation profiles and their clinical significance in poorly elucidated tonsil cancers, with the highest count of HPV and SCC sites among studies on oropharyngeal cancers in the era of precision medicine. Our data showed a similar pattern of mutation frequencies to TCGA tonsil cancer data; the latter was derived from a smaller patient cohort than that considered in our study. Because tonsil cancer data are very scarce even in the TCGA head and neck cancer database, our findings may be valuable for further understanding the genomic mutation profile of cancers of the tonsils, a specialized oropharyngeal organ among head and neck structures.

## 5. Conclusions

In conclusion, the present study revealed that oncogenic/likely oncogenic mutations are relatively high in tonsil cancers, and that *TP53* and RTK pathway mutations may be candidate predictors for detrimental prognosis in patients receiving adjuvant therapy and for overall tonsil cancer patients/HPV-positive subgroup, respectively, according to the AJCC eighth edition staging system. The findings of this study may pave the way for further prospective investigations and may be clinically relevant for the potential personalized therapeutic options for tonsil cancer.

## Figures and Tables

**Table 1:** Baseline characteristics of patients with tonsillar squamous cell carcinoma.

Variable	Total
	n = 43 (%)
Sex	
Male	38 (88.4)
Female	5 (11.6)
Age (mean, range)	54 (36–80)
Age (y)	
≤60	28 (65.1)
>60	15 (34.9)
Smoking history	
Low	21 (48.8)
Heavy	22 (51.2)
Alcohol history	
Light	27 (62.8)
Heavy	16 (37.2)
HPV status	
Negative	8 (18.6)
Positive	35 (81.4)
pT category	
1	9 (20.9)
2	13 (30.25)
3	13 (30.25)
4	8 (18.6)
pN category	
0	8 (18.6)
1	18 (41.9)
2	8 (18.6)
3	9 (20.9)
AJCC stage (8th)	
I	15 (34.9)
II	8 (18.6)
III	7 (16.3)
IV	13 (30.2)
PD-L1 expression	
Negative	33 (76.7)
Positive	10 (23.3)
Adjuvant therapy	
Done	28 (65.1)
Not done	15 (34.9)

HPV, human papillomavirus; AJCC, American Joint Committee on Cancer; PD-L1, programmed death-ligand 1.

**Table 2:** Genetic alterations in coding sequences in the context of tonsillar squamous cell carcinoma.

Pathways	Genes	No. Variant Calls (%)	No. Cases (%)
EGFR pathway		39 (32.5)	22 (51.2)
	*EGFR*, *ERBB2*		
	*KRAS*, *HRAS*		
	*PIK3CA*, *PTEN*, *AKT1*		
p53 pathway (genome integrity)			21 (48.8)
	*TP53*	30 (25.0)	20 (46.5)
	*ATM*	2 (1.7)	2 (4.7)
PI3K pathway			17 (39.5)
	*PIK3CA*	13 (10.8)	11 (25.6)
	*PTEN*	9 (7.5)	8 (18.6)
	*AKT1*	1 (0.8)	1 (2.3)
RTK pathway			15 (34.9)
	*KIT*	3 (2.5)	3 (7.0)
	*PDGFRA*	4 (3.3)	4 (9.3)
	*EGFR*	9 (7.5)	7 (16.3)
	*ERBB2*	2 (1.7)	2 (4.7)
	*FGFR2*	2 (1.7)	2 (4.7)
	*FGFR3*	1 (0.8)	1 (2.3)
	*MET*	1 (0.8)	1 (2.3)
	*RET*	1 (0.8)	1 (2.3)
	*FLT3*	1 (0.8)	1 (2.3)
	*ALK*	2 (1.7)	2 (4.7)
RB pathway (cell cycle)			8 (18.6)
	*RB1*	5 (4.2)	5 (11.6)
	*CDKN2A*	3 (2.5)	3 (7.0)
TGF-β pathway	*SMAD4*	8 (6.8)	6 (14.0)
MAPK pathway			5 (11.6)
	*KRAS*	2 (1.7)	2 (4.7)
	*HRAS*	3 (2.5)	3 (7.0)
Proteolysis	*FBXW7*	5 (4.2)	5 (11.6)
SWI/SNF complex	*SMARCB1*	4 (3.3)	4 (9.3)
Notch pathway	*NOTCH1*	2 (1.7)	2 (4.7)
HIPPO pathway	*CDH1*	2 (1.7)	2 (4.7)
mTOR pathway	*STK11*	1 (0.8)	1 (2.3)
Metabolism	*IDH1*	1 (0.8)	1 (2.3)
	*IDH2*	1 (0.8)	1 (2.3)
Transcription factor/regulator	*VHL*	1 (0.8)	1 (2.3)
Other	*MPL*	1 (0.8)	1 (2.3)

RTK, receptor tyrosine kinase; PI3K, phosphatidylinositol 3-kinase; EGFR, epidermal growth factor receptor; RB, retinoblastoma tumor suppressor; TGF-β, transforming growth factor-beta; MAPK, mitogen-activated protein kinase; SWI/SNF complex, SWItch/Sucrose Non-Fermentable complex; mTOR, mammalian target of rapamycin.

**Table 3:** Correlation of the mutation status with clinicopathological parameters in tonsillar squamous cell carcinomas.

Parameter	*TP53*			*PIK3CA*			EGFR Pathway			P53 Pathway			PI3K Pathway			RTK Pathway		
	MT	WT	*p*	MT	WT	*p*	MT	WT	*p*	MT	WT	*p*	MT	WT	*p*	MT	WT	*p*
	n = 20 (%)	n = 23 (%)		n = 11 (%)	n = 32 (%)		n = 22 (%)	n = 21 (%)		n = 21 (%)	n = 22 (%)		n = 17 (%)	n = 26 (%)		n = 15 (%)	n = 28 (%)	
Sex			0.051			0.306			0.185			0.048			0.139			0.798
Male	20 (100)	18 (78.3)		11 (100)	27 (84.4)		21 (95.5)	17 (81.0)		21 (100)	17 (77.3)		17 (100)	21 (80.8)		13 (86.7)	25 (89.3)	
Female	0 (0)	5 (21.7)		0 (0)	5 (15.6)		1 (4.5)	4 (19.0)		0 (0.0)	5 (22.7)		0 (0.0)	5 (19.2)		2 (13.3)	3 (10.7)	
Age (yrs)			0.512			0.719			0.526			0.666			0.745			0.235
≤60	12 (60.0)	16 (69.6)		8 (72.7)	20 (62.5)		13 (59.1)	15 (71.4)		13 (61.9)	15 (68.2)		12 (70.6)	16 (61.5)		8 (53.3)	20 (71.4)	
>60	8 (40.0)	7 (30.4)		3 (27.3)	12 (37.5)		9 (40.9)	6 (28.6)		8 (38.1)	7 (31.8)		5 (29.4)	10 (38.5)		7 (46.7)	8 (28.6)	
Smoking			0.033			0.736			0.763			0.015			1.000			0.526
Low	6 (30.0)	15 (65.2)		6 (54.5)	15 (46.9)		10 (45.5)	11 (52.4)		6 (28.6)	15 (68.2)		8 (47.1)	13 (50.0)		6 (40.0)	15 (53.6)	
Heavy	14 (70.0)	8 (34.8)		5 (45.5)	17 (53.1)		12 (54.5)	10 (47.6)		15 (71.4)	7 (31.8)		9 (52.9)	13 (50.0)		9 (60.0)	13 (46.4)	
Alcohol			0.127			0.494			0.537			0.215			1.000			0.782
Light	10 (50.0)	17 (73.9)		8 (72.7)	19 (59.4)		15 (68.2)	12 (57.1)		11 (52.4)	16 (72.7)		11 (64.7)	16 (61.5)		9 (60.0)	18 (64.3)	
Heavy	10 (50.0)	6 (26.1)		3 (27.3)	13 (40.6)		7 (31.8)	9 (42.9)		10 (47.6)	6 (27.3)		6 (35.3)	10 (38.5)		6 (40.0)	10 (35.7)	
HPV			0.017			0.392			0.698			0.021			0.502			0.320
Negative	7 (35.0)	1 (4.3)		3 (27.3)	5 (15.6)		5 (22.7)	3 (14.3)		7 (33.3)	1 (4.5)		4 (23.5)	4 (15.4)		4 (26.7)	4 (14.3)	
Positive	13 (65.0)	22 (95.7)		8 (72.7)	27 (84.4)		17 (77.3)	18 (85.7)		14 (66.7)	21 (95.5)		13 (76.5)	22 (84.6)		11 (73.3)	24 (85.7)	
AJCC stage			0.547			0.736			0.763			0.366			1.000			0.019
I–II	9 (45.0)	13 (56.5)		5 (45.5)	17 (53.1)		12 (54.5)	10 (47.6)		9 (42.9)	13 (59.1)		9 (52.9)	13 (50.0)		4 (26.7)	18 (64.3)	
III–IV	11 (55.0)	10 (43.5)		6 (54.5)	15 (46.9)		10 (45.5)	11 (52.4)		12 (57.1)	9 (40.9)		8 (47.1)	13 (50.0)		11 (73.3)	10 (35.7)	
pT category			0.763			1.000			0.131			1.000			0.537			0.347
T1–T2	11 (55.0)	11 (47.8)		6 (54.5)	16 (50.0)		14 (63.6)	8 (38.1)		11 (52.4)	11 (50.0)		10 (58.8)	12 (46.2)		6 (40.0)	16 (57.1)	
T3–T4	9 (45.0)	12 (52.2)		5 (45.5)	16 (50.0)		8 (36.4)	13 (61.9)		10 (47.6)	11 (50.0)		7 (41.2)	14 (53.8)		9 (60.0)	12 (42.9)	
pN category			0.038 *			0.656			0.412			0.046			0.298			0.391
N0	6 (30.0)	1 (4.3)		1 (9.1)	6 (18.8)		5 (22.7)	2 (9.5)		6 (28.6)	1 (4.5)		4 (23.5)	3 (11.5)		1 (6.7)	6 (21.4)	
N+	14 (70.0)	22 (95.7)		10 (90.9)	26 (81.2)		17 (77.3)	19 (90.5)		15 (71.4)	21 (95.5)		13 (76.5)	23 (88.5)		14 (93.3)	22 (78.6)	
PD-L1			0.011			0.409			0.162			0.069			0.061			1.000
Negative	19 (95.0)	14 (60.9)		10 (90.9)	23 (71.9)		19 (86.4)	14 (66.7)		19 (90.5	14 (63.6)		16 (94.1)	17 (65.4)		12 (80.0)	21 (75.0)	
Positive	1 (5.0)	9 (39.1)		1 (9.1)	9 (28.1)		3 (13.6)	7 (33.3)		2 (9.5)	8 (36.4)		1 (5.9)	9 (34.6)		3 (20.0)	7 (25.0)	

MT, mutant; WT, wild-type; HPV, human papillomavirus; AJCC, American Joint Committee on Cancer; EGFR, epidermal growth factor receptor; RTK, receptor tyrosine kinase; PD-L1, programmed death-ligand 1. * Statistically significant, at *p* < 0.05.P53 pathway mutations were associated with male sex (*p* = 0.048), heavy smoking history *(p* = 0.015), HPV negativity (*p* = 0.021), and node-negative tumors (*p* = 0.046), whereas those in the RTK pathway were strongly associated with advanced stages (*p* = 0.019). However, there were no meaningful correlations between the PI3K pathway mutations and clinicopathological features.

**Table 4:** Univariate and multivariate analyses of factors predicting overall and disease-free survival of patients with tonsillar squamous cell carcinoma.

	Overall Survival				Disease-Free Survival			
	Univariate		Multivariate		Univariate		Multivariate	
	HR (95% CI)	*p*	HR (95% CI)	*p*	HR (95% CI)	*p*	HR (95% CI)	*p*
Sex	1.105	0.894			1.337	0.695		
Female vs. Male	(0.256–4.767)				(0.313–5.710)			
Age (y)	3.146	0.011 *	3.565	0.020 *	2.140	0.072	2.068	0.088
≤60 vs. >60	(1.296–7.639)		(1.221–10.412)		(0.935–4.902)		(0.898–4.761)	
Smoking	1.427	0.433			1.126	0.777		
Low vs. Heavy	(0.587–3.471)				(0.495–2.561)			
Alcohol	1.651	0.272			1.126	0.782		
Light vs. Heavy	(0.675–4.041)				(0.485–2.615)			
HPV	0.385	0.058	1.901	0.313	0.567	0.239		
Negative vs. Positive	(0.144–1.033)		(0.546–6.618)		(0.220–1.460)			
AJCC stage	4.688	0.003 *	5.246	0.006 *	3.386	0.008 *	2.856	0.029 *
I–II vs. III–IV	(1.687–13.030)		(1.596–17.235)		(1.380–8.306)		(1.110–7.345)	
Adjuvant therapy	1.287	0.607			1.736	0.247		
Yes vs. No	(0.493–3.356)				(0.683–4.418)			
*TP53*	2.221	0.088	1.781	0.265	1.436	0.390		
WT vs. MT	(0.887–5.563)		(0.646–4.909)		(0.629–3.280)			
*PIK3CA*	0.787	0.642			0.892	0.811		
WT vs. MT	(0.286–2.166)				(0.351–2.267)			
RTK pathway	3.371	0.007 *	1.798	0.214	2.349	0.042 *	1.584	0.295
WT vs. MT	(1.390–8.178)		(0.712–4.539)		(1.033–5.339)		(0.670–3.746)	
p53 pathway	1.915	0.161			1.248	0.597		
WT vs. MT	(0.772–4.752)				(0.548–2.843)			
PI3K pathway	0.647	0.353			0.816	0.635		
WT vs. MT	(0.258–1.622)				(0.352–1.890)			
EGFR pathway	0.669	0.372			0.781	0.556		
WT vs. MT	(0.277–1.618)				(0.344–1.776)			

HR, hazard ratio; CI, confidence interval; HPV, human papillomavirus; AJCC, American Joint Committee on Cancer; MT, mutant; WT, wild type; RTK, receptor tyrosine kinase; EGFR, epidermal growth factor receptor. * Statistically significant, at *p* < 0.05.

**Table 5:** Univariate and multivariate analyses of factors predicting overall and disease-free survival in patients with HPV-positive tonsil cancer.

	Overall Survival				Disease-Free Survival			
	Univariate		Multivariate		Univariate		Multivariate	
	HR (95% CI)	*p*	HR (95% CI)	*p*	HR (95% CI)	*p*	HR (95% CI)	*p*
Sex	0.658	0.585			0.833	0.809		
Female vs. Male	(0.147–2.953)				(0.190–3.653)			
Age (y)	4.942	0.003 *	10.521	0.002 *	3.165	0.021 *	4.378	0.010 *
≤60 vs. >60	(1.705–14.329)		(2.426–45.616)		(1.190–8.420)		(1.429–13.408)	
Smoking	1.447	0.493			1.159	0.762		
No vs. Current/Former	(0.504–4.159)				(0.446–3.013)			
Alcohol	1.624	0.390			1.086	0.877		
Light vs. Heavy	(0.538–4.907)				(0.381–3.093)			
AJCC stage	5.205	0.006 *	9.576	0.003 *	3.809	0.009 *	4.700	0.007 *
I–II vs. III–IV	(1.622–16.707)		(2.141–42.823)		(1.399–10.370)		(1.520–14.530)	
*TP53*	1.482	0.474			0.972	0.955		
WT vs. MT	(0.505–4.350)				(0.358–2.636)			
*PIK3CA*	0.428	0.268			0.606	0.432		
WT vs. MT	(0.096–1.918)				(0.174–2.113)			
P53 pathway	1.246	0.686			0.821	0.698		
WT vs. MT	(0.428–3.631)				(0.303–2.223)			
PI3K pathway	0.357	0.115			0.587	0.318		
WT vs. MT	(0.099–1.284)				(0.207–1.670)			
RTK pathway	4.314	0.007 *	1.979	0.218	2.758	0.037*	1.415	0.495
WT vs. MT	(1.489–12.502)		(0.667–5.868)		(1.061–7.173)		(0.522–3.836)	
EGFR pathway	0.522	0.247			0.702	0.474		
WT vs. MT	(0.174–1.567)				(0.267–1.849)			

HR, hazard ratio; CI, confidence interval; HPV, human papillomavirus; AJCC, American Joint Committee on Cancer; MT, mutant; WT, wild type; RTK, receptor tyrosine kinase; EGFR, epidermal growth factor receptor. * Statistically significant, at *p* < 0.05.

**Table 6:** Univariate and multivariate analyses of factors predicting overall and disease-free survival of patients with tonsillar squamous cell carcinoma who underwent adjuvant therapy.

	Overall Survival				Disease-Free Survival			
	Univariate		Multivariate		Univariate		Multivariate	
	HR (95% CI)	*p*	HR (95% CI)	*p*	HR (95% CI)	*p*	HR (95% CI)	*p*
Sex	2.616	0.354			3.535	0.221		
Female vs. Male	(0.342–20.020)				(0.468–26.710)			
Age (y)	2.211	0.158			1.490	0.456		
≤60 vs. >60	(0.735–6.648)				(0.522–4.248)			
Smoking	1.429	0.505			1.079	0876		
Low vs. Heavy	(0.500–4.086)				(0.416–2.800)			
Alcohol	2.112	0.185			1.285	0.639		
Light vs. Heavy	(0.698–6.388)				(0.451–3.657)			
HPV	0.340	0.076	1.146	0.841	0.601	0.376		
Negative vs. Positive	(0.103–1.119)		(0.302–4.355)		(0.195–1.856)			
AJCC stage	4.765	0.017 *	4.856	0.024 *	2.868	0.049 *	3.075	0.039 *
I–II vs. III–IV	(1.319–17.220)		(1.228–19.201)		(1.005–8.187)		(1.057–8.944)	
*TP53*	4.582	0.011 *	4.348	0.022 *	2.439	0.075	2.653	0.059
WT vs. MT	(1.419–14.789)		(1.242–15.223)		(0.913–6.511)		(0.963–7.309)	
*PIK3CA*	0.759	0.643			0.871	0.796		
WT vs. MT	(0.237–2.428)				(0.306–2.483)			
P53 pathway	3.678	0.029 *	2.681	0.153	1.929	0.187		
WT vs. MT	(1.144–11.827)		(0.693–10.368)		(0.727–5.117)			
PI3K pathway	0.663	0.463			0.906	0.842		
WT vs. MT	(0.222–1.984)				(0.343–2.391)			
RTK pathway	2.848	0.055			1.653	0.303		
WT vs. MT	(0.980–8.279)				(0.636–4.297)			
EGFR pathway	0.598	0.344			0.745	0.546		
WT vs. MT	(0.206–1.735)				(0.286–1.941)			

HR, hazard ratio; CI, confidence interval; HPV, human papillomavirus; AJCC, American Joint Committee on Cancer; MT, mutant; WT, wild type; RTK, receptor tyrosine kinase; EGFR, epidermal growth factor receptor. * Statistically significant, at *p* < 0.05.

## Data Availability

The data used to support the findings of this study are available from the corresponding author upon request.

## Supplementary Materials

The following supporting information can be downloaded at https://www.mdpi.com/article/10.3390/biomedicines11030851/s1, Table S1: Full list of pathogenic mutations.
