# Supplementary material for: Assessment of the Mutation Profile of Tonsillar Squamous Cell Carcinomas Using Targeted Next-Generation Sequencing
Source: Biomedicines. 2023 Mar 10;11(3):851. doi: 10.3390/biomedicines11030851 (PMC10045642; doi:10.3390/biomedicines11030851)
Supplement: Supplementary file 1 [file biomedicines-11-00851-s001.zip › biomedicines-2237878-supplementary.pdf]

Supplementary Table S1. Full list of pathogenic mutations

Hugo_Symbol	Chromosome	Position	Ref	Alt	Variant_Classification	HGVSp_Short	Coverage	Alt count	Frequency	Sample Name	HPV
PIK3CA	chr3	178936091	G	A	Missense_Mutation	p.E545K	1997	143	7.2	225_26_s606_8256	pos
TP53	chr17	7578235	T	C	Missense_Mutation	p.Y205C	1999	157	7.9	225_26_s606_8256	pos
NOTCH1	chr9	139399362	C	T	Missense_Mutation	p.R1594Q	349	23	6.6	274_29_S715_10256	pos
TP53	chr17	7579442	G	-	Frame_Shift_Del	p.P82Rfs*41	590	211	35.8	274_29_S715_10256	pos
RB1	chr13	48942685	C	T	Nonsense_Mutation	p.R358*	187	14	7.5	257_31_s610_6032	pos
IDH1	chr2	209113112	C	T	Missense_Mutation	p.R132H	1797	154	8.6	227_9_S606_6278	pos
CDKN2A	chr9	21971155	G	A	Missense_Mutation	p.A68V	919	109	11.9	227_9_S606_6278	pos
TP53	chr17	7578458	G	A	Missense_Mutation	p.R158C	907	74	8.2	227_9_S606_6278	pos
STK11	chr19	1220487	G	A	Missense_Mutation	p.D194N	1505	78	5.2	227_9_S606_6278	pos
SMARCB1	chr22	24133967	C	T	Nonsense_Mutation	p.R40*	506	77	15.2	227_9_S606_6278	pos
PIK3CA	chr3	178936091	G	A	Missense_Mutation	p.E545K	1998	158	7.9	210_18_02_4428	pos
PIK3CA	chr3	178936082	G	A	Missense_Mutation	p.E542K	950	196	20.6	207_21_01_6107	pos
ERBB2	chr17	37881332	G	A	Missense_Mutation	p.V842I	400	39	9.8	207_21_01_6107	pos
SMAD4	chr18	48591918	C	T	Missense_Mutation	p.R361C	107	18	16.8	207_21_01_6107	pos
PDGFRA	chr4	55152108	C	T	Missense_Mutation	p.S847L	181	151	83.4	236_22_S607_3808	neg
FGFR2	chr10	123279677	G	A	Missense_Mutation	p.S252L	1197	169	14.1	236_22_S607_3808	neg
FLT3	chr13	28592624	T	C	Missense_Mutation	p.N841D	981	73	7.4	236_22_S607_3808	neg
TP53	chr17	7578458	G	A	Missense_Mutation	p.R158C	1056	415	39.3	236_22_S607_3808	neg
AKT1	chr14	105246482	C	T	Missense_Mutation	p.E40K	812	100	12.3	214_24_03_5248	neg
TP53	chr17	7578212	G	-	Frame_Shift_Del	p.R213Dfs*34	1057	144	13.6	214_24_03_5248	neg
TP53	chr17	7578212	G	A	Nonsense_Mutation	p.R213*	1226	317	25.9	214_24_03_5248	neg
SMAD4	chr18	48575152	C	T	Nonsense_Mutation	p.Q116*	2000	180	9	214_24_03_5248	neg
PTEN	chr10	89720799	TACT	-	Frame_Shift_Del	p.T319*	1395	327	23.4	217_25_04_6322	pos
PDGFRA	chr4	55152108	C	T	Missense_Mutation	p.S847L	1081	78	7.2	226_27_s606_7629	pos

EGFR	chr7	55241726	C	T	Missense_Mutation	p.T725M	2000	108	5.4	226_27_s606_7629	pos
TP53	chr17	7577090	C	T	Missense_Mutation	p.R283H	353	18	5.1	226_27_s606_7629	pos
TP53	chr17	7578211	C	T	Missense_Mutation	p.R213Q	1566	114	7.3	226_27_s606_7629	pos
TP53	chr17	7578235	T	C	Missense_Mutation	p.Y205C	1567	89	5.7	226_27_s606_7629	pos
TP53	chr17	7578464	G	A	Missense_Mutation	p.R156C	1997	100	5	226_27_s606_7629	pos
SMAD4	chr18	48593507	C	T	Missense_Mutation	p.R420C	210	22	10.5	226_27_s606_7629	pos
SMAD4	chr18	48604665	G	A	Missense_Mutation	p.R496H	1054	69	6.5	226_27_s606_7629	pos
SMAD4	chr18	48604668	G	A	Missense_Mutation	p.R497H	1054	69	6.5	226_27_s606_7629	pos
VHL	chr3	10188200	C	T	Missense_Mutation	p.H115Y	83	21	25.3	243_29_S608_8707	pos
FGFR3	chr4	1803565	G	A	Missense_Mutation	p.R248H	1999	174	8.7	243_29_S608_8707	pos
PTEN	chr10	89624232	AGCCATCATCAAAGAGATCGTT	-	Frame_Shift_Del	p.I4Efs*13	2855	838	29.4	222_30_05_8684	pos
TP53	chr17	7578470	CGGG	-	Frame_Shift_Del	p.P153Afs*16	1974	1182	59.9	223_31_05_6348	pos
PTEN	chr10	89692905	G	A	Missense_Mutation	p.R130Q	299	18	6	272_4_S708_9684	pos
PTEN	chr10	89692911	G	A	Missense_Mutation	p.G132D	309	18	5.8	272_4_S708_9684	pos
ALK	chr2	29432664	C	T	Missense_Mutation	p.R1275Q	267	150	56.2	231_17_S607_9963	neg
PIK3CA	chr3	178936091	G	A	Missense_Mutation	p.E545K	1196	264	22.1	231_17_S607_9963	neg
PIK3CA	chr3	178952141	G	A	Missense_Mutation	p.A1066T	153	13	8.5	231_17_S607_9963	neg
NOTCH1	chr9	139399351	G	A	Missense_Mutation	p.R1598C	488	121	24.8	231_17_S607_9963	neg
PTEN	chr10	89624288	T	C	Missense_Mutation	p.F21S	601	35	5.8	231_17_S607_9963	neg
TP53	chr17	7578544	G	A	Missense_Mutation	p.A129V	2000	217	10.8	231_17_S607_9963	neg
SMARCB1	chr22	24143240	C	T	Nonsense_Mutation	p.R158*	295	84	28.5	231_17_S607_9963	neg
RET	chr10	43615578	G	A	Missense_Mutation	p.R886Q	196	15	7.7	249_19_S609_5250	pos
TP53	chr17	7577138	C	T	Missense_Mutation	p.R267Q	204	13	6.4	249_19_S609_5250	pos
PTEN	chr10	89711899	C	T	Missense_Mutation	p.R173C	233	12	5.2	250_20_S609_5496	pos
TP53	chr17	7577547	C	T	Missense_Mutation	p.G245D	1980	582	29.4	250_20_S609_5496	pos
EGFR	chr7	55242511	G	A	Missense_Mutation	p.D761N	485	76	15.7	213_21_03_8350	pos
PTEN	chr10	89711900	G	A	Missense_Mutation	p.R173H	186	16	8.6	213_21_03_8350	pos

RB1	chr13	49033844	C	T	Missense_Mutation	p.R661W	209	11	5.3	213_21_03_8350	pos
IDH2	chr15	90631935	G	A	Missense_Mutation	p.R140W	310	40	12.9	213_21_03_8350	pos
TP53	chr17	7578461	C	T	Missense_Mutation	p.V157I	1886	98	5.2	213_21_03_8350	pos
TP53	chr17	7578469	C	A	Missense_Mutation	p.G154V	1781	1350	75.8	213_21_03_8350	pos
SMAD4	chr18	48575159	C	T	Missense_Mutation	p.A118V	753	39	5.2	213_21_03_8350	pos
FBXW7	chr4	153247288	C	T	Missense_Mutation	p.R505H	2000	230	11.5	253_22_S609_11232	pos
FGFR2	chr10	123279677	G	A	Missense_Mutation	p.S252L	1205	95	7.9	253_22_S609_11232	pos
KRAS	chr12	25398284	C	T	Missense_Mutation	p.G12D	707	335	47.4	253_22_S609_11232	pos
TP53	chr17	7577570	C	T	Missense_Mutation	p.M237I	1997	119	6	253_22_S609_11232	pos
TP53	chr17	7578217	G	A	Missense_Mutation	p.T211I	819	41	5	253_22_S609_11232	pos
TP53	chr17	7578289	C	A	Missense_Mutation	p.G187V	815	97	11.9	253_22_S609_11232	pos
TP53	chr17	7579359	G	A	Missense_Mutation	p.R110C	210	112	53.3	253_22_S609_11232	pos
ERBB2	chr17	37881010	C	T	Missense_Mutation	p.P780L	1587	94	5.9	253_22_S609_11232	pos
PIK3CA	chr3	178936091	G	A	Missense_Mutation	p.E545K	1995	103	5.2	216_24_04_8528	neg
TP53	chr17	7578235	T	C	Missense_Mutation	p.Y205C	1467	109	7.4	216_24_04_8528	neg
PIK3CA	chr3	178936091	G	A	Missense_Mutation	p.E545K	1993	260	13	244_30_S608_9308	pos
PDGFRA	chr4	55152071	T	C	Missense_Mutation	p.C835R	978	49	5	246_32_S609_2328	pos
EGFR	chr7	55233037	C	T	Missense_Mutation	p.P596L	1939	245	12.6	246_32_S609_2328	pos
TP53	chr17	7578422	T	C	Missense_Mutation	p.T170A	140	32	22.9	246_32_S609_2328	pos
FBXW7	chr4	153249385	G	A	Missense_Mutation	p.R465C	112	14	12.5	264_1_S09_9525	pos
KIT	chr4	55599332	G	T	Missense_Mutation	p.D820Y	488	33	6.8	268_2_S08_2402	pos
CDH1	chr16	68835629	C	T	Nonsense_Mutation	p.R74*	1158	80	6.9	268_2_S08_2402	pos
PIK3CA	chr3	178928079	G	A	Missense_Mutation	p.E453K	107	16	15	271_3_S06_2164	pos
PTEN	chr10	89624254	A	G	Missense_Mutation	p.S10G	408	33	8.1	271_3_S06_2164	pos
RB1	chr13	49027168	C	T	Nonsense_Mutation	p.R579*	76	10	13.2	271_3_S06_2164	pos
TP53	chr17	7578457	C	T	Missense_Mutation	p.R158H	224	80	35.7	271_3_S06_2164	pos
PIK3CA	chr3	178916936	G	A	Missense_Mutation	p.R108H	407	41	10.1	228_10_S606_5581	neg

PIK3CA	chr3	178921548	G	A	Missense_Mutation	p.V344M	1554	101	6.5	228_10_S606_5581	neg
KIT	chr4	55594197	C	T	Missense_Mutation	p.R634W	527	60	11.4	228_10_S606_5581	neg
FBXW7	chr4	153259007	G	A	Nonsense_Mutation	p.Q270*	447	26	5.8	228_10_S606_5581	neg
EGFR	chr7	55249071	C	T	Missense_Mutation	p.T790M	709	38	5.4	228_10_S606_5581	neg
CDKN2A	chr9	21971063	G	A	Missense_Mutation	p.R99W	1264	84	6.6	228_10_S606_5581	neg
ATM	chr11	108236229	G	A	Nonsense_Mutation	p.W3055*	1369	90	6.6	228_10_S606_5581	neg
TP53	chr17	7579455	C	-	Frame_Shift_Del	p.A78Qfs*45	633	132	20.9	228_10_S606_5581	neg
SMAD4	chr18	48591918	C	T	Missense_Mutation	p.R361C	751	136	18.1	228_10_S606_5581	neg
FBXW7	chr4	153250883	G	A	Nonsense_Mutation	p.R393*	1766	121	6.9	230_12_S607_10592	pos
KRAS	chr12	25378561	G	C	Missense_Mutation	p.A146G	234	26	11.1	230_12_S607_10592	pos
RB1	chr13	48955538	C	T	Nonsense_Mutation	p.R552*	784	152	19.4	230_12_S607_10592	pos
TP53	chr17	7577103	C	-	Frame_Shift_Del	p.R280Efs*65	188	13	6.9	230_12_S607_10592	pos
TP53	chr17	7577102	C	A	Missense_Mutation	p.G279V	213	23	10.8	230_12_S607_10592	pos
TP53	chr17	7578449	C	-	Frame_Shift_Del	p.A161Pfs*9	1437	194	13.5	230_12_S607_10592	pos
HRAS	chr11	533874	T	C	Missense_Mutation	p.Q61R	685	219	32	209_17_02_3803	pos
SMAD4	chr18	48593507	C	T	Missense_Mutation	p.R420C	219	13	5.9	209_17_02_3803	pos
PIK3CA	chr3	178936082	G	A	Missense_Mutation	p.E542K	1997	630	31.5	232_18_S607_9225	pos
TP53	chr17	7577539	G	A	Missense_Mutation	p.R248W	256	30	11.7	232_18_S607_9225	pos
EGFR	chr7	55249017	C	T	Missense_Mutation	p.P772L	236	14	5.9	212_20_03_6924	pos
MET	chr7	116417445	C	T	Missense_Mutation	p.H1106Y	473	24	5.1	212_20_03_6924	pos
HRAS	chr11	533874	T	C	Missense_Mutation	p.Q61R	569	49	8.6	212_20_03_6924	pos
SMARCB1	chr22	24176338	C	T	Missense_Mutation	p.R377C	435	33	7.6	212_20_03_6924	pos
PDGFRA	chr4	55152062	G	T	Missense_Mutation	p.V832L	445	37	8.3	239_25_S608_4692	neg
FBXW7	chr4	153249384	C	T	Missense_Mutation	p.R465H	473	200	42.3	239_25_S608_4692	neg
EGFR	chr7	55241678	A	G	Missense_Mutation	p.E709G	2000	105	5.2	239_25_S608_4692	neg
EGFR	chr7	55249028	C	T	Missense_Mutation	p.R776C	807	68	8.4	239_25_S608_4692	neg
CDKN2A	chr9	21971197	ATCAT	-	Frame_Shift_Del	p.M53Gfs*65	1937	426	22	239_25_S608_4692	neg

TP53	chr17	7578421	G	A	Missense_Mutation	p.T170M	1008	230	22.8	239_25_S608_4692	neg
MPL	chr1	43815021	C	T	Missense_Mutation	p.A519V	1628	99	6.1	241_27_S607_4120	pos
RB1	chr13	49033845	G	A	Missense_Mutation	p.R661Q	595	69	11.6	241_27_S607_4120	pos
TP53	chr17	7579359	G	A	Missense_Mutation	p.R110C	612	121	19.8	241_27_S607_4120	pos
SMARCB1	chr22	24176339	G	A	Missense_Mutation	p.R377H	355	67	18.9	241_27_S607_4120	pos
PIK3CA	chr3	178936091	G	A	Missense_Mutation	p.E545K	1998	130	6.5	242_28_S607_3732	pos
EGFR	chr7	55249007	G	A	Missense_Mutation	p.V769M	768	63	8.2	242_28_S607_3732	pos
EGFR	chr7	55259545	A	G	Missense_Mutation	p.E868G	887	48	5.4	242_28_S607_3732	pos
PTEN	chr10	89720871	T	C	Missense_Mutation	p.F341S	255	23	9	242_28_S607_3732	pos
HRAS	chr11	534286	C	T	Missense_Mutation	p.G13S	1010	80	7.9	242_28_S607_3732	pos
ATM	chr11	108172385	C	T	Nonsense_Mutation	p.R1730*	874	274	31.4	242_28_S607_3732	pos
CDH1	chr16	68835629	C	T	Nonsense_Mutation	p.R74*	2000	151	7.5	242_28_S607_3732	pos
PIK3CA	chr3	178936082	G	A	Missense_Mutation	p.E542K	1997	578	28.9	245_31_S609_503	pos
ALK	chr2	29443643	G	A	Missense_Mutation	p.R1192W	312	27	8.7	224_32_05_2023	pos
KIT	chr4	55594198	G	A	Missense_Mutation	p.R634Q	92	14	15.2	224_32_05_2023	pos
